# Bifid Rib With an Unusual Appearance on Axial Computed Tomography

**DOI:** 10.1002/ccr3.73692

**Published:** 2026-10-05

**Authors:** Yuya Okuyama, Tsuyoshi Nojima, Kohei Tokioka, Atsunori Nakao

**Affiliations:** ^1^ Department of Emergency, Critical Care, and Disaster Medicine, Faculty of Medicine, Dentistry and Pharmaceutical Sciences Okayama University Okayama Japan

**Keywords:** bifid rib, chest pain, computed tomography, congenital rib anomaly, multiplanar imaging

## Abstract

When a rib appears focally elongated on axial CT, a bifid rib should be considered. Contiguous axial and multiplanar review can clarify this benign congenital anatomy and help avoid misinterpreting it as a pathological osseous lesion.

A 23‐year‐old woman was brought to the emergency department by ambulance because of worsening left‐sided chest pain that had begun the day before her presentation. The pain was sharp, localized to the lateral chest wall, and exacerbated by deep inspiration and movement. She reported no history of trauma, fever, cough, or dyspnea. Physical examination revealed no swelling, subcutaneous emphysema, rash, or focal tenderness of the chest wall. Breath sounds were symmetric, without wheezes or crackles. Cardiac examination revealed a regular rhythm with no murmurs. Electrocardiography showed sinus rhythm, and serum cardiac troponin levels were negative.

Because the cause of the chest pain remained unclear after the initial evaluation, non–contrast‐enhanced chest CT was performed using a 64‐row multidetector CT scanner (SOMATOM go. Top, Siemens Healthineers, Forchheim, Germany). The axial images used for the initial interpretation were reconstructed with a section thickness of 1 mm. On axial CT, the anterior portion of the left fourth rib showed an unusual focal appearance that initially suggested apparent elongation. However, review of contiguous axial images and coronal reformatted images demonstrated a flat, plate‐like bifurcated bony structure with preserved cortex and no fracture, cortical disruption, or destructive change (Figure 1A,B). Three‐dimensional reconstruction was used to visually demonstrate the bifurcated morphology of the left fourth rib (Figure 1C). The rib anomaly was considered an isolated congenital variant rather than an acute pathological finding responsible for the patient's symptoms. The patient was treated with nonsteroidal anti‐inflammatory drugs for presumed musculoskeletal chest wall pain, and the symptoms gradually improved.

**FIGURE 1:**
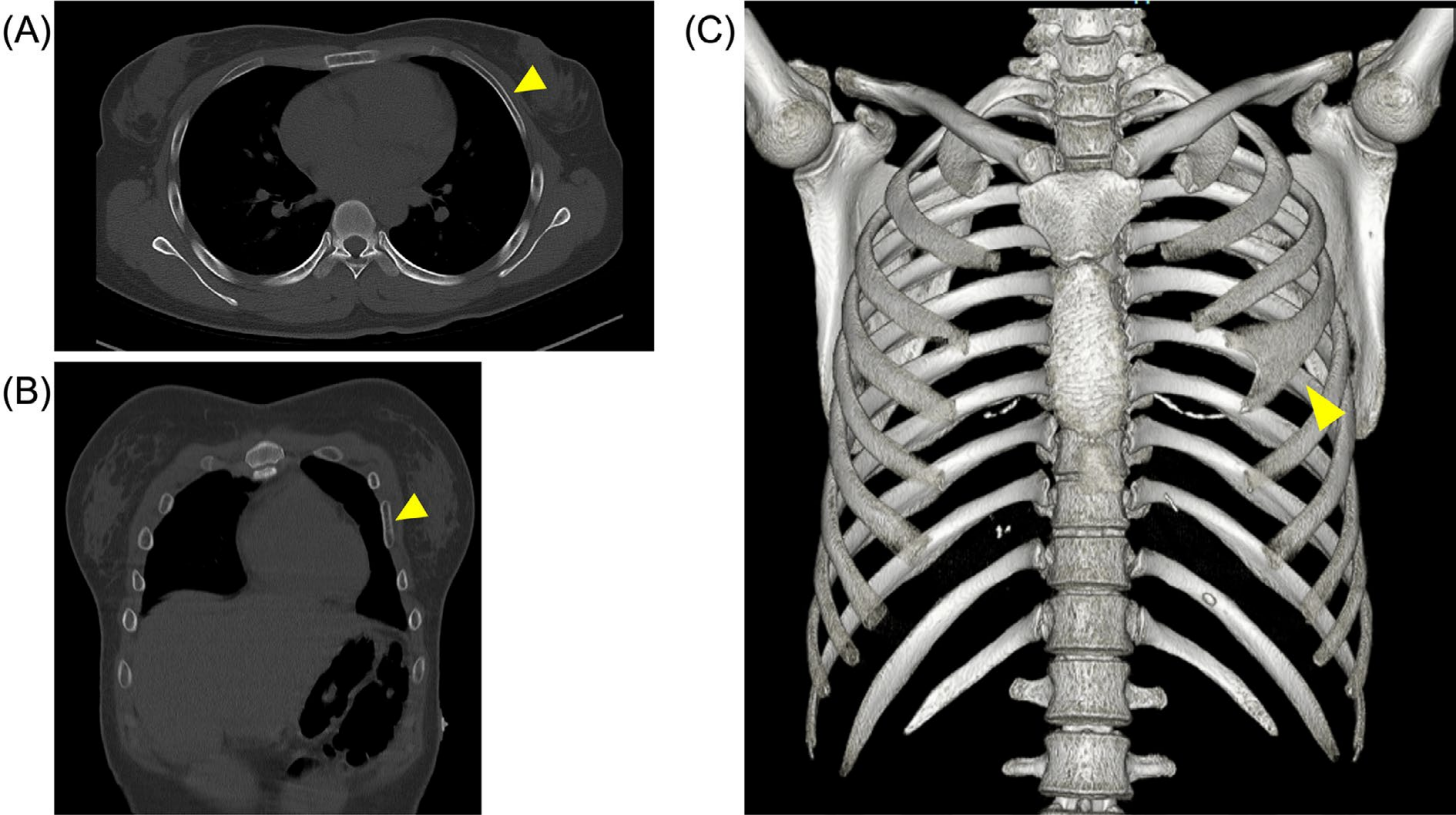
(A) Axial and (B) coronal computed tomography images of the chest demonstrating the different appearances of the left fourth rib. On axial imaging, the rib appeared elongated, whereas coronal reformatted images showed a flat, plate‐like configuration without cortical disruption or destructive changes. (C) Three‐dimensional reconstruction demonstrating bifurcation of the left fourth rib (arrow), confirming a bifid rib.

Congenital rib anomalies are often incidentally detected on thoracic imaging [1]. A bifid rib is a congenital variant characterized by the partial duplication of the rib, most commonly involving the anterior portion of the fourth rib [1]. It is generally asymptomatic and does not require intervention when it is isolated [2]. However, depending on the imaging plane, its morphology may produce an unusual focal appearance on CT [1, 2].

The key teaching point in this case is that an atypical focal rib appearance on axial CT should be reconciled with contiguous axial images and multiplanar reformatted images before being interpreted as a pathological osseous lesion. In this patient, coronal reformatted images clarified the plate‐like bifurcated morphology of the left fourth rib, while three‐dimensional reconstruction provided an intuitive visual demonstration of the congenital anatomy. This case illustrates the educational value of correlating discordant appearances across axial, coronal, and three‐dimensional images.

Awareness of congenital rib variants, such as bifid ribs, may help clinicians interpret unusual rib morphologies more appropriately in clinical practice, particularly when evaluating chest CT obtained for acute symptoms.

## Author Contributions

**Kohei Tokioka:** writing – review and editing. **Yuya Okuyama:** investigation, writing – original draft. **Tsuyoshi Nojima:** conceptualization, project administration, visualization, writing – review and editing. **Atsunori Nakao:** writing – review and editing, supervision.

## Funding

The authors have nothing to report.

## Ethics Statement

The authors have nothing to report.

## Consent

Written informed consent was obtained from the patient for the publication of this case report, including the images.

## Conflicts of Interest

The authors declare no conflicts of interest.

## Data Availability

Data sharing is not applicable to this article, as no datasets were generated or analyzed during the current study.
